# Navigating Power Imbalances and Stigma in Mental Healthcare. Patient‐Reported Barriers and Facilitators to Participation in Shared Decision‐Making in Mental Health Care, a Qualitative Meta‐Summary

**DOI:** 10.1111/hex.70239

**Published:** 2025-04-07

**Authors:** Lien Mertens, Joris Vandenberghe, Geertruida Bekkering, Karin Hannes, Nicolas Delvaux, Pieter Van Bostraeten, Jasmien Jaeken, Bert Aertgeerts, Mieke Vermandere

**Affiliations:** ^1^ Department of Public Health and Primary Care KU Leuven Leuven Belgium; ^2^ Psychiatry Research Group, Department of Neurosciences KU Leuven UPC KU Leuven Leuven Belgium; ^3^ Belgian Centre for Evidence‐Based Medicine Leuven Belgium; ^4^ Research Group SoMeTHin'K, Faculty of Social Science, KU Leuven Leuven Belgium; ^5^ Belgium; JBI Belgium: A JBI Affiliated Group Leuven Belgium; ^6^ Department of Public Health and Primary Care Campus Kortrijk (KULAK) ‐ KU Leuven Belgium

**Keywords:** barriers and facilitators, mental healthcare, patient participation, patient perspective, patient‐reported, shared decision‐making

## Abstract

**Background:**

The use of shared decision‐making (SDM) in mental healthcare has been viewed as at least as important as its use in non‐mental healthcare settings, but it still does not routinely take place in this setting. To further explore SDM processes with people with lived experience, we provide a qualitative meta‐summary on patient‐reported barriers and facilitators to participation in SDM within the context of mental healthcare.

**Methods:**

Within the set of selected studies for a larger qualitative meta‐summary, using five databases, we selected the studies that had surveyed patients with mental illness for further analysis in this paper. Search terms were based on the concepts: ‘decision making’, ‘patient participation’, ‘patient perceptions’ and ‘study design’ of patient reporting, including patient surveys, interviews and focus groups.

**Results:**

Out of the 90 studies that had been selected for the larger review, we selected 13 articles concerning mental illness for more detailed analysis in this review. In total, we identified 29 different influencing factors and we found 6 major barriers: ‘Lack of choice’, ‘Not being respected as a person’, ‘Feeling stigma from physician’, ‘Disease burden’, ‘Power imbalance’ and ‘Low self‐efficacy to participate’. ‘Clear information provision about options’, ‘Being respected as a person, being taken into account’, ‘Good physician‐patient relationship’ and ‘Belief in the importance of one's own role’ were the main facilitators.

**Conclusions:**

Stigma and self‐stigma still seem to persist in mental healthcare and continue to suppress patients' self‐efficacy to participate in SDM in this setting. There is much discussion of inclusion and diversity worldwide, and these themes are just as topical for patients with mental health problems. Further work seems necessary to eradicate all stigma and self‐stigma in this setting when striving for care that could be ‘as shared as possible’.

**Patient and Public Contribution:**

The authors wish to thank Mr. Walter Geuens, a person with lived experience in mental healthcare, for his careful reading and thorough feedback on the final paper.

## 1 Background

Shared decision‐making (SDM) is defined as ‘an approach where clinicians and patients share the best available evidence when faced with the task of making treatment decisions, and where patients are supported in considering treatment options and achieving informed preferences’ [1, 2, 3]. Although SDM is now internationally seen as an ethical imperative [1, 4, 5], a hallmark of good clinical practice and the ‘pinnacle of patient‐centred care’ [6] and included as a fundamental patient right in a growing number of national health care standards and legislation including Belgium [7, 8, 9, 10, 11], it is still not widely implemented in clinical care [12]. Nor does SDM routinely occur in mental healthcare [13, 14, 15].

However, the use of SDM in mental health care has been viewed as at least as important as its use in non‐mental healthcare settings because of the lack of a specific test or scan to diagnose and treat ailments in mental healthcare [16]. The information on how someone is feeling about what they have experienced informs the process of how physicians and therapists make treatment decisions, thus increasing the reliance on patient involvement in mental healthcare [16]. Additionally, the principles of SDM fit in perfectly with the pragmatic solutions and patient‐specific decisions that are often needed in mental healthcare, especially when treating severe disorders, where fine‐tuned communication skills, patient empowerment and personalised recovery‐oriented care are vital elements [17].

Gaining insight into patients' perspectives on barriers and facilitators to SDM is a valuable strategy to better tailor SDM approaches to the target group's needs and improve overall SDM implementation. Within our larger review, which explored patients' experienced barriers and facilitators to SDM across diverse patient groups and healthcare settings [18], we uncovered ‘common ground’ in the patient perspective across the diversity of groups and settings. However, throughout our analysis, we also uncovered particularities in specific settings, such as the setting of mental healthcare, that would benefit from further attention and analysis. Since none of the latest reviews on patient‐reported barriers and facilitators to participation in SDM [19, 20, 21, 22, 23, 24, 25, 26, 27] contained a specific analysis of the views of (adult) patients with mental illness, an updated, setting‐ and population‐specific overview seemed warranted. Therefore, we chose to complement the insights of our larger review with a more in‐depth analysis of a selection of studies concerning mental healthcare to better identify existing barriers and facilitators to participation in SDM within this particular setting.

We hope this review may further inform future SDM interventions that can support the patients' ability to better participate in SDM in mental healthcare and that can help patients navigate existing challenges.

## 2 Methods

### 2.1 Study Design

This study was part of a larger review on patient‐reported barriers and facilitators to participation in SDM across diverse patient groups and healthcare settings, including an analysis of 90 articles in total [18]. Out of these 90 studies, we selected the studies that had specifically surveyed patients with mental illness for a more in‐depth analysis in this paper. We conducted this synthesis in accordance with the Preferred Reporting Items for Systematic Review and Meta‐Analyses Protocols (PRISMA‐P Checklist, 2015). The research protocol was registered in PROSPERO (https://www.crd.york.ac.uk/prospero/#recordDetails) with ID number CRD42022360313.

### 2.2 Search Strategy and Selection Criteria

We conducted a systematic search using five databases that were searched from January 2012 to April 2023: MEDLINE via PubMed, Cumulative Index of Nursing and Allied Health Literature (CINAHL), EMBASE, Scopus and Web of Science Core Collection. Studies needed to be published in 2012 or later, as the previous broad review on patient‐reported barriers and facilitators to SDM [19] included articles up to August 2012. We adapted our search strategy based on this previous review [19]. Search terms were based on the concepts of ‘decision making’, ‘patient participation’, ‘patient perceptions’ and ‘study design’ of patient reporting, including patient surveys, interviews and focus groups in particular. For this paper, we deliberately chose not to add search terms linked to the context of mental healthcare or mental illness to our original search string so as not to restrict our search. Yet, as mentioned above, we chose to make a selection of studies with a focus on the mental healthcare setting out of the set that had been sampled for the larger qualitative meta‐summary (Appendix—Methodology). To guarantee that we captured all the relevant and most recent literature, we regularly updated our search throughout the process.

### 2.3 Eligibility Criteria

#### 2.3.1 Types of Participants

‘Patients’ were considered the participants of interest. In this paper, the term ‘patient’ specifically refers to anyone who is a ‘user of *mental* healthcare’. ‘Mental healthcare’ was broadly defined as (all) ‘services devoted to treating and supporting people with mental illnesses or impairments’, both including inpatient and outpatient services and including the treatment of severe as well as less severe mental disorders. For further details, we refer to the Appendix.

#### 2.3.2 Types of Outcomes

We considered studies evaluating ‘patient perceptions (attitudes, perspectives, experiences, expectations)’ of barriers and facilitators to involvement in the medical decision‐making process in the context of mental healthcare.

#### 2.3.3 Types of Studies

We included empirical, peer‐reviewed journal articles that used qualitative or mixed‐methods designs, such as patient surveys, (semi‐)/(un‐)structured interviews, focus groups or work groups. Studies exploring multiple stakeholder perspectives (e.g., patients', family members' and/or physicians' perspectives) were included on the condition that the data originating from patients could be distinguished from other stakeholders' views, as only the data originating from patients were intended to be analysed further in this paper.

### 2.4 Study Identification and Data Extraction

We removed duplicates in Endnote (Version 20) using Bramer's de‐duplication procedure [28]. Then, the de‐duplicated search results were integrated into the review software programme Covidence and screened independently and in duplicate by six reviewers on title and abstract (M.L., S.R., K.T., J.J., V.B.P. and A.B.) and next by two reviewers on full text (M.L. and K.T.). Reasons for exclusion of full‐text papers were recorded and reported in the PRISMA flow chart [29]. Any disagreements were resolved through discussion within the team.

Study characteristics of all eligible articles were collected in descriptive data extraction sheets in Excel. We extracted the primary author, year of publication, country of origin, aim, methodology, sample size, healthcare setting (community, primary, secondary or tertiary care) and disease setting. Quotes and descriptions originating from the text under the headings ‘results/conclusions’ of the included studies were extracted and thematised into barriers/facilitators in other data extraction sheets.

### 2.5 Theme Analysis

We analysed and reported the findings in several formats because of the complexities inherent to the topic. First, we constructed a detailed taxonomy of barriers and facilitators based on earlier taxonomies on patient‐reported barriers and facilitators to SDM [19, 20, 21, 22, 30, 31]. We primarily used a *deductive analysis approach* to identify the first set of barriers/facilitators, as we built on insights from earlier literature [19], including our own review [18]. Yet, we allowed (and even actively sought for) additional dimensions to emerge during analysis. Secondly, we created an overview of all barriers and facilitators in order of reporting frequency to identify the factors that were most frequently recurring. Thirdly, we clustered the most frequently recurring barriers/facilitators into broader analytical themes, reflecting patients' most common challenges and longings with regard to participation in SDM in this setting. Lastly, we created a figure to offer a visual summary of those most frequently recurring barriers and facilitators and deepened our findings in a narrative report below, supported by illustrative quotes.

## 3 Results

### 3.1 Included Studies

We primarily found 9265 unique references and withheld 209 eligible studies after screening. Out of the set of 209 eligible studies, 90 were sampled and analysed in the larger review [18]. Out of this sample (*n* = 90), 13 articles regarding mental healthcare were selected for more detailed analysis in this review (Flow chart 1, 1).

**Flow chart 1:**
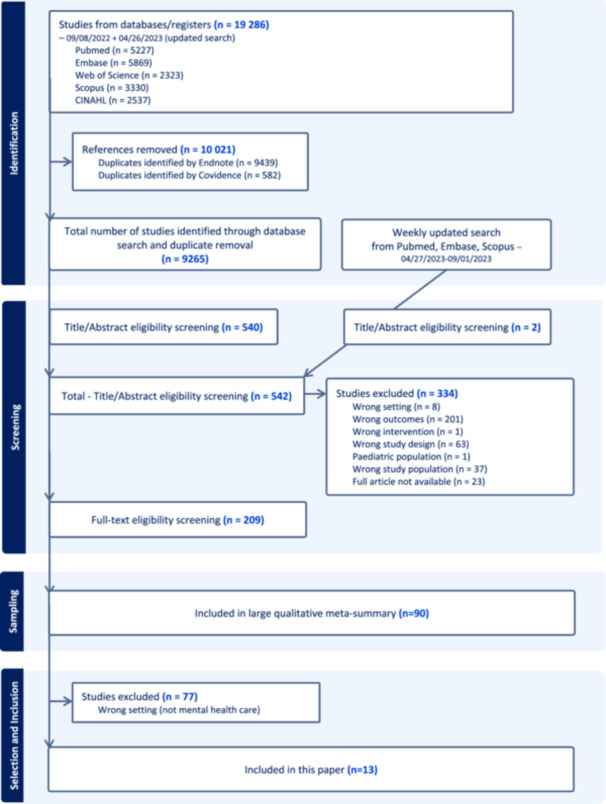
Flow diagram of the inclusion process of relevant papers

To guarantee that we captured the most recent insights, we updated our search a final time in Medline on 22 March 2024. This led to 502 papers, including 4 more about SDM in mental healthcare and 1 more eligible article [32]. However, since this latter article did not reveal any new themes compared to those we had already identified in the original set of articles (*n* = 13), it was not added to this review. Study characteristics (*n* = 13) are included in Table 1.

**Table 1:** Study characteristics (*n* = 13).

Reference	Author	Year	Country	Aim	Methodology	Sample size	Healthcare setting, disease setting
[33]	Aoki	2019	Japan	To explore patients' experiences of an SDM programme developed for psychiatric outpatients who faced new treatment decisions.	Qualitative, semi‐structured interviews	10	Psychiatric outpatient clinic, psychiatric illness
[34]	Beyene	2019	Norway	To interpret the meaning of shared decision‐making in mental care as perceived by patients and mental healthcare professionals.	Qualitative, in‐depth interviews (patients), multistage focus group interviews (HCP)	16	Community mental health centre, psychiatric illness
[35]	Carrotte	2021	Australia	To identify facilitators and barriers associated with shared decision‐making in Australians affected by schizophrenia spectrum disorders.	Mixed‐methods, surveys and interviews	6	Psychiatry, schizophrenia spectrum disorder
[36]	Eliacin	2015	United States	To investigate how patients with mental illness understand the concept of shared decision‐making.	Qualitative, semi‐structured interviews	54	Outpatient mental healthcare, psychiatric illness
[37]	Eliacin	2015	United States	To investigate patients' preferences and appraisals of their involvement in treatment decisions.	Qualitative, interviews	54	Outpatient mental healthcare, psychiatric illness
[38]	Dahlqvist Jönsson	2015	Sweden	To explore users' experiences of participation in decisions in mental health services in Sweden and the kinds of support that may promote participation.	Qualitative, focus groups, individual interviews	20	community, psychiatric illness—diverse diagnoses
[39]	Delman	2015	United States	To explore young adult perceptions of the barriers and facilitators to active participation in psychotropic medication decisions.	Qualitative, cross‐sectional interviews	24	Community and clinical psychiatric settings, ‘serious mental illness’
[40]	Hamann	2016	Germany	To explore both patients' and psychiatrists' views on how patients can facilitate shared decision‐making in acute mental health settings.	Qualitative, focus groups	16	Psychiatry, schizophrenia, depression, bipolar disorder
[41]	Kivelitz	2018	Germany	To investigate the information needs and the decision‐making preferences of patients with mental disorders before the decision for a certain treatment setting.	Qualitative, Semi‐structured interviews	24	Psychotherapy inpatient hospital setting, psychiatric illness
[42]	Kokanovic	2018	Australia	To explore from several perspectives the barriers and facilitators to supported decision‐making in an Australian context.	Qualitative—narrative interviews	29	Mental health community, psychiatric illness—diverse diagnoses
[16]	Maples	2022	United States	To identify the factors influencing the use of a shared decision‐making model in a transitional care clinic providing treatment to people with serious mental illness after the occurrence of a psychiatric crisis or hospitalisation.	Qualitative, semi‐structured interviews	15	Psychiatry, ‘Serious mental illness’
[43]	Matthews	2021	United States	To explore patients' experiences of SDM and articulate communication and decision‐making preferences among an underserved patient population receiving depression treatment in an urban, safety net primary care clinic.	Mixed‐methods—survey and semi‐structured interviews	27	Primary care clinic, depression
[44]	Rodenburg	2020	Netherlands	To investigate patients' and clinicians' perspectives on SDM to treat depression, anxiety disorders and OCD so as to better understand SDM in specialised psychiatric care and its challenges in clinical practice.	Qualitative, focus groups	17	Psychiatry, depression, anxiety disorder and OCD

### 3.2 Themes

#### 3.2.1 Theme Analysis

In total, we identified 29 different influencing factors, acting either as barrier (*n* = 14), facilitator (*n* = 14) or barrier or facilitator to an equal extent (*n* = 1). Table 2a offers a detailed taxonomy. Tables 2b, 2c and 2d give an overview of all influencing factors, in order of reporting frequency, and the most frequently discussed aspects per factor.

**Table 2a:** Taxonomy of barriers and facilitators (B = barrier, F = facilitator, B/F = mentioned both as barrier and facilitator).

Patient‐related factors	Interactional factors	Physician‐related factors	Environmental and organisational factors
Individual ‐ Feeling informed *Clear information provision about options (F)* *To educate and prepare yourself as a patient (F)* ‐ Expectations and attitudes *No or few notions of SDM (B)* *Seeing the physician as ‘the expert’ (B)* *Physicians' explicit invitation or encouragement to participate (F)* *Ideas concerning the ‘good patient’ (B)* ‐ Self‐awareness *Low self‐efficacy to participate (B)* *Belief in the importance of one's own role and active participation (F)* ‐ Preferences *Wish for restriction of information (B)* *Physicians' and patients' honesty (F)* *Low motivation to participate (B)* Decision ‐ Illness *Disease burden (B)* *Timing of decision (later in life or disease course) (F)* ‐ Options *Lack of choice (B)*	Relationship ‐ Patient–clinician relationship *Patient–physician relationship (F)* *Trust in physician (B/F)* ‐ Patient–clinician communication *Not being respected as a person, not being taken into account (B)* *Being respected as a person, being taken into account (B)* *Power imbalance (B)* *Feeling stigma from the physician (B)* ‐ Social support *Support of family/friends/peers (F)* *Support of other healthcare providers (F)*	Physicians' time *Physicians' limited time (B)* *Physicians' time (F)* Physicians' skills ‐ Explaining skills *Clear information provision about options (F)* ‐ Listening skills *Being respected as a person, being taken into account (F)* ‐ Training *Training of healthcare providers (F)*	Healthcare context ‐ Care pathway *Discontinuity of care (B)* *Time to think (F)* *(Unfavourable) setting/resources (B)*

**Table 2b:** Overview of barriers in order of reporting frequency (*n* = 14).

Barriers	Most frequently discussed aspects	Frequency of reporting	
		n/13	%
1. Lack of choice (B)	Physicians push for one option, unbalanced information and offer only one choice.	11	85
2. Not being respected as a person, not being taken into account (B)	Not feeling listened to, not feeling to be taken seriously, authentic connection, experiencing no interest of physician in the patient, disrespect, feeling ignored or experiencing dismissive behaviours.	7	54
3. Feeling stigma from the physician (B)	Experiencing suspicion from physicians, feeling of not being taken seriously, feeling ignored or judged as less capable than others, physicians being judgemental.	7	54
4. Disease burden (B)	Feeling lost, loss of sense of competence, feeling not able (cognitively) to process information or to contribute, reduced desire to participate.	7	54
5. Power imbalance (B)	Asymmetric power relationship, dominance of physician, patients' competences and equality not taken into account, physicians being patronising, physicians' reluctance to involve patients in decisions.	6	46
6. Low self‐efficacy to participate (B)	Patients' lack of belief in one's own capacity, undervaluing of own expertise.	6	46
7. Physicians' limited time (B)	Limited physicians' time as a barrier to discuss and reflect, to give your opinion (as a patient), lengthy waiting times as a barrier to establishing a useful relationship, time pressure resulting in physicians appearing bothered and rushed.	5	38
8. Seeing the physician as the expert (B)	Superior expertise of physicians, undervaluing own expertise, physicians' choice is always right/the best.	4	31
9. Discontinuity of care (B)	Poor continuity of care as a barrier to establishing a useful relationship and collaboration.	3	23
10. (Unfavourable) setting/resources (B)	Out‐of‐pocket costs, lengthy waiting lists, lack of availability of physicians.	3	23
11. Ideas concerning the ‘good patient’ (B)	Not wanting to trouble the physician or being considered a ‘difficult’ patient, believing you have to comply as a patient, believing it as not appropriate to object your physician, wanting to avoid conflict.	2	15
12. No/few notions of SDM (B)	Not being aware that there are decisions that one may be involved with, seeing one's role in the decision as limited to accepting or rejecting the offered procedure, lacking awareness of the possibility to choose or the option to refuse treatment, unfamiliarity with SDM concept.	2	15
13. Low motivation to participate (B)	Due to mental illness and reduced overall motivation, feeling a few need to be involved.	2	15
14. Wish for restriction of information (B)	Because of disease burden (‘head filled’ with mental issues), ‘too much’ information is experienced as a burden. Less information (and less involvement) is thus preferred.	1	8

**Table 2c:** Overview of facilitators in order of reporting frequency (*n* = 13).

Facilitators	Most frequently discussed aspects	Frequency of reporting	
		n/13	%
1. Clear information provision about options (F)	Longing for (more) information, sufficiency and transparency, physician's responsibility, importance of written or visual format or support of decision aids.	9	69
2. Being respected as a person, being taken into account (F)	(The importance of) feeling listened to, taken into account, taken seriously, experiencing an authentic connection, physicians' genuine interest and respect.	8	62
3. Good physician–patient relationship (F)	Good rapport with physician, mutual respect, warm connection and longevity of relationship.	7	54
4. Belief in the importance of one's own role and active participation (F)	Patients' belief in one's own capacity, belief in importance of participation, importance of assertiveness, speaking up, daring to ask and informing oneself.	6	46
5. To educate and prepare yourself as a patient (F)	Information‐seeking behaviour, studying of options in (peaceful) home environment, preparation of questions before consultation and feeling more confident to enter discussion with physician.	**5**	**38**
6. Physicians' explicit invitation/encouragement to participate (F)	Expecting the physician to actively encourage patients to participate and express their opinions, explicitly ask their input, expecting to be invited to participate as an approvement for involvement and not sharing any information if not explicitly asked to do so.	5	38
7. Support of family, friends and peers (F)	Decisional support from family or significant others; those ‘who know patients well’ and ‘who matter most’ because they are ‘well‐informed of their health needs’, (online) peer support, the value of others' experiences, (one papers) family involvement as challenging, e.g., causing difficulties in discussing sensitive information (mental health as a ‘private domain’)	5	38
8. Time to think (F)	Time to reflect and make decisions, time to discuss with others and time to absorb the information.	3	23
9. Physicians' and patients' honesty (F)	Expecting honesty from physicians, not wanting to be withheld information, even if burdensome (risks or other). Need to be honest as a patient to disclose symptoms and side effects.	3	23
10. Timing of decision (later in life or disease course) (F)	Growth in confidence and assertiveness along the disease or life course (because of growth in life or disease experience).	3	23
11. Physicians' time (F)	Positive effects of physicians ‘taking’ their time, (offering) time to discuss and reflect as a patient.	2	15
12. Support of other healthcare providers (F)	Support of ‘support staff’, a ‘support’ person, therapist or other.	2	15
13. Assertiveness	Speaking openly to physicians, (daring to) ask(ing) questions and (daring to) express(ing) your preferences.	2	15
14. Training of healthcare providers (F)	Communication training and training to take away stereotyping and stigmatising by physicians.	1	8

**Table 2d:** Overview of influencing factors that were discussed as barriers and facilitators to equal extent, in order of reporting frequency (*n* = 1).

Influencing factors (Facilitator ⇔ Barrier)			
Trust in physician (B/F)	Blind trust, superior expertise of physicians, physicians acting in patients' best interest, trust because of long relationship and trust as facilitating/stimulating participation.	6	46

**Figure 1:**
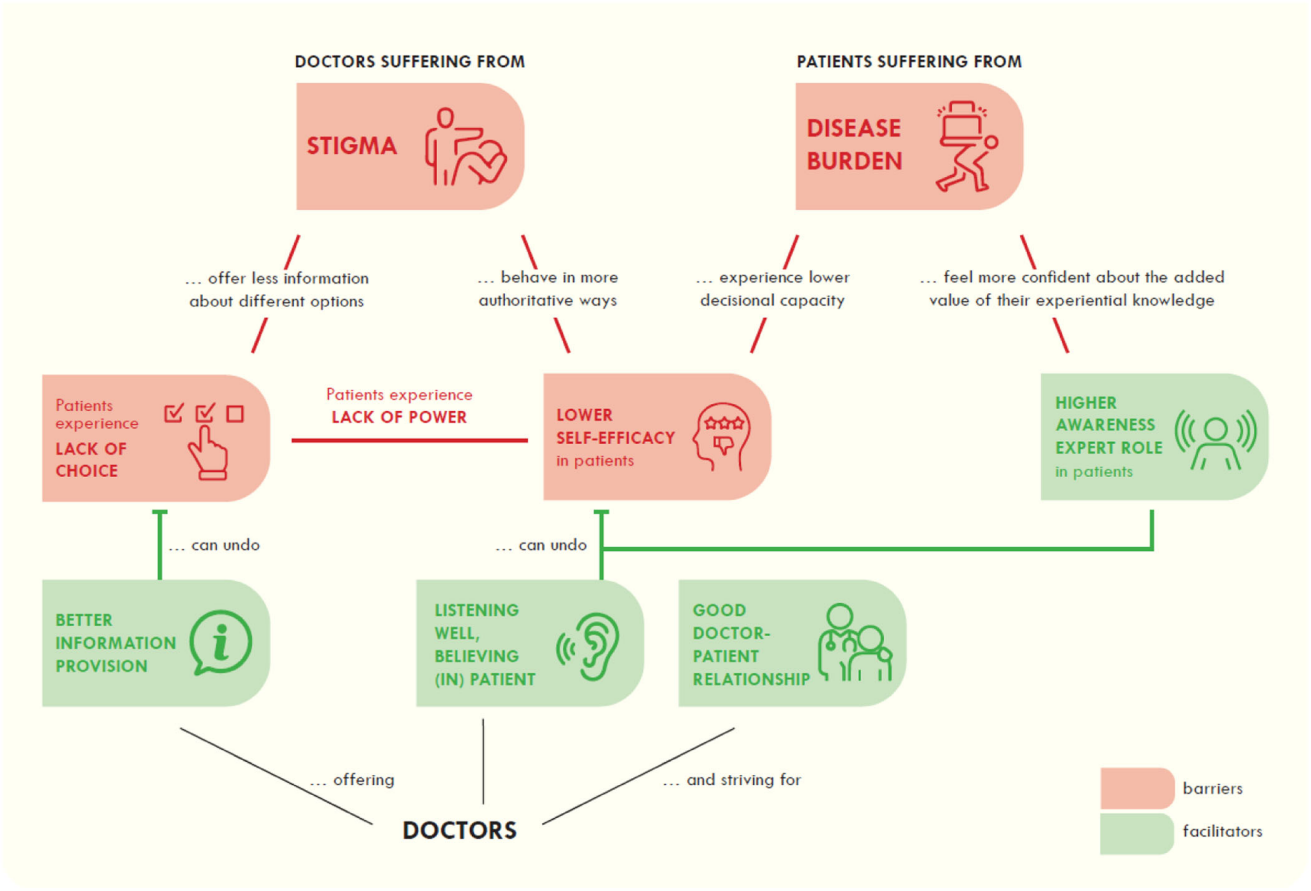
Infographic of most frequently reported influencing factors and their interconnectedness.

Building on the most frequently reported influencing factors, we defined three overarching analytical themes, reflecting patients' most frequently recurring challenges and longings in this setting. The narrative report below textually elaborates on these. Table 3 in the Appendix offers illustrative quotes for some intensively discussed influencing factors. Figure 1, 1 represents an infographic of the most frequently reported influencing factors and their interconnectedness.

##### 3.2.1.1 Theme 1: Mental illness and SDM: two sides of a coin

**Table 3:** Illustrative quotes for intensively discussed influencing factors.

Intensively discussed influencing factors	Illustrative quotes
1 Clear information provision about options (F)	*‐ ‘(…) I think it's good to be informed as much as possible about the existing options and get involved into the decision‐making process’* [41] *‐ ‘(the patient) needs information from the clinician (to decide)’* [41] *‐ ‘patients preferred to be well‐informed’* [44] *‐ ‘respondents expressed feeling that since they had less knowledge, their prospects for actively participating in care decisions (…) was reduced.’* [38]
2. Lack of choice (B)	*‐ ‘not feeling in a position to participate when they did not have the whole picture.’* [38] *‐ ‘I just didn't know I could choose which medications I could be on or that I could refuse at all.’* [39] *‐ ‘[…] I just didn't know it was my decision.’* [39]
3. Stigma (B)	*‐ ‘(…) history of substance use created (…) suspicion for providers, which in turn affected the patient‐provider relationship and SDM’* [37] *‐ ‘they look at me like you're just wanting to get high’* [37] *‐ ‘I feel like whenever I talk to them about what worked for me, they look at me like you're just wanting to get high’* [26] *‐ ‘I had absolutely no influence; they gave me the tablets and said: Open your mouth…’* [40] *‐ ‘[…] she just persuaded me and I had no choice.’* [40] *‐ ‘They don't even care. They just give you these prescriptions, thank you, bye‐bye.’* [16] *‐ ‘[…] the attitude of some physicians like “just take what I told you don't ask questions”.’* [43]
4. Disease burden (B)	*‐ ‘Many patients emphasized that mental illness often reduces their desire to participate in decision‐making (…) depressive symptoms in particular’* [40] *‐ ‘Being depressed I did not want to know about anything and also did not want to talk to any doctor (…) I did not want to participate.’* [40] *‐ ‘When I'm very ill I don't always know what's best for me.’* [34] *‐ ‘If the psychiatrist had asked me to decide only by myself, I would not have known how to decide and would have been at a loss.’* [33] *‐ ‘In situations where the patients needed the health professionals to assist them in decision‐making, but the professionals provided them the responsibility to decide for themselves and act independently, the patient felt rather helpless and insignificant.’* [34] *‐ ‘(…) due to my anxiety disorder, I was scared a lot. In that case I think it helps when the doctor says: “Do this, do that.”’* [41]
5. Belief in the importance of one's own role and active participation (F)	‐ *‘Having a question or wanting detailed information is possibly annoying but important*.*’* [6]
6. (Not) being respected as a person and (not) being taken into account (F)	*‐ ‘The next psychiatrist I saw, I felt more her equal…I felt like there was more negotiation with this particular doctor. I felt like she listened better and she cared about me rather than just medicating me.’* [35] *‐ ‘provider's communication skills (…) helped them to open up and seek the care they needed’* [36] *‐ ‘I feel comfortable enough with her that I can open up.’* [36] *‐ ‘I now say something, where before I wouldn't have said much.’* [39] *‐ ‘I was getting to know him and started to feel more comfortable talking about side effects as they occurred.’* [39]

The presence of mental illness and its impact on patients' feelings regarding participation in SDM seemed to be ambivalent. On one hand, many patients described experiencing a ‘*disease burden’* (7/13; 54%) because of mental illness, which was perceived to have an important, negative impact on patients' decisional capacity (‘*Low self‐efficacy to participate’*, 6/13; 46%) and motivation to be involved in decisions (2/13; 15%). On the other hand, the presence of mental illness, the (often) chronic nature of it and the associated build‐up of illness experiences helped many patients to feel more confident about the added value of their contribution and the importance of their active participation in SDM (‘*Belief in the importance of one's own role and active participation’*; 6/13; 46%).

###### 3.2.1.1.1 Disease Burden

Many patients felt that their ability to participate in SDM was reduced when suffering from mental illness. First, they felt that their *cognitive* ability to understand and process information was easily disturbed when experiencing a high burden of mental disorder [41, 44]. They felt that psychiatric symptoms, especially when experiencing an acute exacerbation of them, could even render them cognitively (totally) ‘*unable*’ to be actively involved in the decision‐making process [16, 35, 38, 44].

Besides the cognitive impact, many patients experienced increased emotions because of mental illness, such as doubts, fears, feelings of ‘*helplessness*’ [41] and indecisiveness in the process [16, 34], further reducing their self‐confidence to participate. Patients described experiencing a loss of their ‘*inner sense of competence’* [41], a loss of *‘trust in their own judgment’* [44] and ‘*insight into their situation’* [34] because their psyche was *‘so frail’* [41]. They reported that they ‘*would not have known how to decide’*, ‘*would have been at a loss’* or would have felt *‘helpless and insignificant*’ if their physician had given them ‘*the responsibility to decide for themselves and act independently’* [33, 34]. Patients also reported that when *‘your head is [already] filled with all of that [mental concerns]’* [41], ‘*getting even more on top’* [41] in the form of an active decision‐making role was not always desirable. ‘*Too much information’* was experienced as ‘*a burden’* since ‘*dealing with your head’* is already ‘*burden enough’* and ‘*makes everything harder’* [41]. Mental space for reasoning was often impacted by the burden of mental illness and further decreased patients' motivation to be involved [40].

‘He [the physician] knows better than me. […] Who am I?’ [16]

Because of these challenges, some patients felt more satisfied with the idea that the clinician would (sometimes) *‘take the lead’* in the decision‐making process [41, 44] and would offer them the option of adopting a more passive decision‐making role [41], mostly in cases of high disease burden. In case of fear for their own safety or in case of a lack of insight into the situation, patients preferred physicians to adopt an ‘*authoritarian style’* to safeguard safety and dignified care [16, 34]. Illness severity was thus considered an ‘*important factor in successfully incorporating SDM into psychiatric care*’ [44].

‘Both too much and too little autonomy could threaten the patients' dignity, depending on the patients' mental health.’ [34]

###### 3.2.1.1.2 Experiential Expertise

Despite the major challenges, patients also saw opportunities in being mentally ill, such as the build‐up of ‘experiential knowledge’ and expertise. Many patients in this setting confidently reported that they believe they are ‘*very experienced’*, ‘*know their body’*, are ‘*their own expert’* on disease and symptoms and ‘*know what's best for them’* [16, 40], rendering patient involvement ‘*important and self‐evident’* [44]. Considering oneself ‘*the boss’* of their own body [44], they sensed a ‘*need*’ [40], and (sometimes) even a *‘responsibility’* [37], to participate in the decision‐making process.

However, they described that they also want their physicians to acknowledge the value of their contribution, to view people with lived experience as ‘*having expertise’* (too) [35] and to consider ‘*their [patients'] competences and equality in relation to the professional’* (more frequently) [36, 38], regardless of the presence of mental illness [35]. They explicitly made clear that they want their physicians to be ‘*flexible with goals, needs and choices’* [35] and take patients' opinions and health goals into consideration, even if it would mean taking a decision that is ‘*opposed to what providers believe is best practice*’, such as reducing or stopping treatment [16].

‘They should listen more to the patient before they come up with solutions to things they don't know!’ [34]

##### 3.2.1.2 Theme 2: Stigma of physicians: restricting patient choice and contribution

Unfortunately, patients in this setting did not (always) feel recognised in their expert role and did not (always) feel ‘choice’ was even presented to them. On the contrary, experiencing a ‘*lack of choice*’ (11/13; 85%) was the most frequently described barrier in this setting, followed by experiences of ‘*not being respected as a person, not being taken into account’* (7/13; 54%), ‘*feeling stigma from physician*’ (7/13; 54%) and ‘*power imbalance*’ (6/13; 46%), further impacting patients' (low) self‐efficacy to participate (6/13; 46%).

###### 3.2.1.2.1 Restricting Patient Choice

Many patients reported having struggled to be seen as ‘*a competent and equal person*’ [38] and having felt judged as *‘less capable than others*’ [35, 42] or as ‘*not having the decision‐making capacity’* (at all) ‘*despite being well’* [35]. The ‘*lack of choice’* patients experienced in this setting seemed to be one of the most explicit expressions of this judgement.

Patients often did not feel sufficiently informed about the different options [41, 42], had regularly experienced ‘*inequalities in access to knowledge’* compared to professionals and had experienced how these inequalities constituted an important barrier to participation [38]. Patients often felt that physicians had offered options in an *‘insufficient’* [41], *‘unbalanced’*, ‘*biased’* or *‘constrained’* way [42, 44], which had made them feel *‘steered*’ [44] or *‘controlled*’ by physicians [38]. They described how they had experienced *‘the physician's own treatment preferences [often] determined the options offered to patients’* [44] and how it had often felt like ‘*the decision […] was already made’* [41] before they had contributed, leaving ‘*little room for alternative suggestions’* [34]. In some cases, the (total) lack of information about alternatives had led to ‘*choice unawareness’* in patients, leaving them completely helpless to contribute [39].

###### 3.2.1.2.2 Restricting Patient Contribution

Some patients recalled even more explicit examples of ‘(*memories of) coercion’* [42], having felt that their point of view was (totally) excluded [38] or ignored [42] and that their ‘*autonomy was rejected’* [34]. Patients described examples of providers' decisions that had been *‘pushed down patients' throats’* [36, 40, 43] and of having had ‘*no choice’, ‘no influence’* [40], *‘no partnership’* [16], *‘no input on anything’* and *‘no power’* [16] (at all) in the decision‐making process. They felt that they had ‘*not been listened to or taken seriously*’ when decisions were made [38] or had even felt treated in a ‘*patronising’, ‘dismissive’* or ‘*judgmental’* way [35].

Such physicians, being *‘rigid, unavailable or distant’* [34] or engaging in ‘*interactions that were perceived as impersonal’* [42], often negatively impacted patients' motivation and abilities to speak up and be involved. Patients were more likely to feel ‘*devalued*’, *‘unappreciated’* and ‘*incompetent’* when they experienced stigma and experienced increased feelings of ‘*powerlessness’* [38]. Stigma often led to *decreased self‐confidence* among patients in their abilities and possibilities to influence their treatment [38, 42] and a ‘*deterrent to express their ideas and preferences’* [40], with effects sometimes continuing ‘*even many years later’* [40]. Such experiences of (very) ‘*poor communication’* [37] and lacking offer of choice thus often amplified feelings of decisional incapacity in patients, further hindering their participation.

Some patients actively reported their frustration around this stigma [35, 38] and wished that psychiatric disorders could be ‘*destigmatised’* and that ‘*a less stereotype‐informed approach’* [35] could be offered to them to enable them to engage (more) in SDM.

##### 3.2.1.3 Theme 3: Patients' longings: greater information flows and good partnerships

###### 3.2.1.3.1 Greater Information Flows

Patients in the included records clearly and recurrently expressed a high need and motivation to be ‘well‐informed’ to participate adequately in the decision‐making process [34, 38, 41, 44]. On one hand, they described the task of information provision as a ‘*responsibility’* of the clinician [44] and stated that they expected him [or her] to ‘*share his expertise’* about options in a way that is ‘*accessible and understandable’* to the patient [43]. On the other hand, becoming educated ‘*through personal efforts*’ in information gathering and accessing resources was thought to further help understand mental illness and feel more confident to actively participate [35, 39]. Physicians complementing their verbal explanation with written or visual information, whether or not in the form of a decision aid, was considered particularly helpful to support patients in accessing the information at home, considering the options in more detail [16, 33, 38, 41] and preparing for discussions with physicians [40].

###### 3.2.1.3.2 Good Partnerships

Patients also recurrently stressed that they ‘*long’* to experience (more) ‘*equality*’ [35, 36, 38] and ‘*partnership*’ [16, 35] in patient–physician interactions through acts of ‘*respect’*, *‘being heard’*, ‘*being understood’*, being ‘*believed’* and ‘*taken seriously’* by the physician [16, 34, 36, 38], to facilitate their participation. They emphasised that they want to be recognised as a *‘(real) person’* [42, 43, 44] and to be viewed as *‘more than a diagnosis’* [43] through the search for an ‘*authentic, interpersonal connection*’ [43] and physicians' act of ‘*genuinely caring’* for and about them as human beings [36, 42, 43]. This approach could help patients to feel (more) ‘*dignified*’, and gain feelings of *safety*, *trust* and feeling *‘cared for’* [16, 34, 38]. It could also help them to feel (more) recognised as *‘able to participate*’ and boost their self‐confidence, motivating them to participate more actively and to ‘*grow’* through these experiences, further boosting their decision‐making capacity and confidence [38, 42].

Lastly, this sense of partnership was thought to help build a ‘*strong’, ‘trusting’* and *‘respectful and caring’* physician–patient relationship [34, 36, 39, 43, 44], which was considered to be ‘*an important prerequisite’* for SDM [44] and even as ‘*integral’* to the process of sharing treatment decisions [36] because of its crucial facilitative effects on patients' comfort in opening up [36, 37, 39]. The importance of being heard and experiencing connection was considered all the more important in the context of high experienced disease burden, vulnerability and uncertainty, as supported by the quote below.

‘When I'm very ill I don't always know what's best for me, but it means a lot to be heard.’ [34]

## 4 Discussion

### 4.1 General Findings

‘*Clear information provision about options’* and ‘*Being respected as a person, being taken into account*’ appeared to be the most frequently reported facilitators overall, suggesting that patients consider these elements to constitute the basis for successful participation in SDM. Yet, opposite experiences of a ‘*lack of choice*’, *‘stigma’* and ‘*power imbalance’* often seem to negatively affect patients' self‐efficacy to participate in this setting. Patients' mental illness creates a challenging ‘*disease burden’*, but also a unique experiential expertise. Patients' awareness of the importance of active participation in SDM and this expert role seems to be relatively high but still underrecognised by physicians. Feeling (more) recognised in the expert role, feeling ‘heard’, ‘believed’ and ‘taken seriously’ and feeling well‐connected with the physician were thus frequently stressed as important facilitators to more successfully engage in SDM in this setting.

### 4.2 Findings Compared to Earlier Literature

Sharing decisions in the setting of mental healthcare is particularly challenging, largely because of the disease burden patients are suffering from. The huge (experienced) impact of mental illness on patients' decision‐making (in)capacity and (dis)empowerment in the process has been described by many previous authors in the field [45, 46, 47, 48, 49, 50, 51, 52, 53, 54, 55]. Patients with mental illness themselves also appear to far more often report their ‘*disease burden*’ as a barrier to participation in SDM, compared to patients suffering from physical illness [18]. As described earlier, psychiatric symptoms, such as lack of insight, delusions and social withdrawal, may directly impede relationships and patients' desire and (perceived) capacity to participate in decisions [56]. Moreover, the possible impact of mental illness on patients' decision‐making (in)capacity has led SDM researchers to question the appropriateness of SDM in this setting. Recent literature on the applicability of SDM defined SDM as ‘not applicable’ or only ‘partially applicable’ in the case of decisions ‘*that may impact patients' or public safety’*, *‘such as in the case of suicidal patients or starting antipsychotic treatment in acutely psychotic patients’* [57, 58]. Considering the complex patient–clinician dynamics, moral dilemmas and social implications of decisions in mental healthcare [17], and, as mentally ill patients' safety may, indeed, be threatened due to lack of (illness) insight, disease symptoms and/or impaired decision‐making capacity, the applicability of SDM is often considered more doubtful than in other healthcare settings. Earlier literature defined that SDM in mental healthcare comes with boundaries and that ‘occasional paternalism’ or physician‐led approaches are sometimes more opportune or even crucial for securing ‘the best care possible’ in this setting [17]. It was suggested that ‘*providers must take clear, honest and transparent positions if they think a shared approach is not within reach’* [17]. However, boundaries in clinical reality are often vague, dynamic and difficult to define, which makes it challenging to take clear positions. The ambiguous offer of choice patients in our data often described might be a reflection of the challenges physicians experience(d) in taking a position and defining the appropriateness and boundaries of SDM in this setting. However, besides the complex impact of the mental disease burden itself, the impact of the stigma associated with mental illness and, especially, of ‘*self‐stigma’* on patients' self‐confidence and on the dynamics within the decision‐making process is important to add, as it differs from patient experiences in other healthcare settings [18]. Several authors have described how physicians often display ambivalent attitudes towards people with mental illness, how they have negative implicit attitudes towards the mentally ill [59] and how these attitudes may cause ‘negative countertransference’ and create, among many other factors, self‐stigma in mentally ill patients [53, 60, 61, 62, 63]. Due to this, patients may feel disempowered and prevented from engaging in SDM, prompting their preference for a more paternalistic decision style, which, in turn, may further fuel their self‐stigma [53, 60, 61, 62, 63]. In the records included in this review, patients never explicitly used the word ‘*self‐stigma’*, but we strongly suspect this phenomenon to be present in the testimonials, as clear testimonials of stigma were encountered and described. With hindsight, it seems difficult to untangle the reported effects of disease burden, stigma and the more subliminally present effects of self‐stigma, on patients' self‐perceived decision‐making capacity.

*Clear information provision about options* (re)appeared as the most frequently reported facilitator, just like in our larger review [18], and in an earlier, broad review on the same topic [19]. This seems to confirm that patients (still) tend to put a lot of emphasis on the *medical information* provided within the SDM process and that this has not changed over the past decades [19]. Moreover, our larger review suggests that this is a patient expectation that is largely irrespective of the healthcare setting or patient group interrogated [18]. The earlier‐identified issue of a remaining focus on the ‘*biological approach’* and on (medical) ‘information provision’ in the SDM process [19, 64, 65], reflected in the (still ongoing) exploding development of decision aids [19], could possibly explain patients' shared expectations and focus on the informational aspect in SDM. However, whereas the ‘*understandability*’ of information was the most frequently emphasised issue within the topic of information provision in our larger review [18], experiencing a *‘lack of choice’* appeared as the most frequently encountered issue related to information provision in the setting of mental healthcare.

### 4.3 Practice Implications

Irrespective of the healthcare setting, intrinsic to the model of treatment, is respect for an individual's *legal capacity*, being the right to make decisions about their treatment and life [66]. Not offering a (full) choice to patients, even with good or well‐founded intentions, always risks creating or maintaining power imbalances between patients and physicians and offending patients' legal right to autonomy and SDM [11]. Because of the changing symptoms and fluctuating course of mental illness, it was suggested that (mentally ill) patients' decisional incapacity should be considered rather ‘a state’ than ‘a trait’ [67] and that SDM would be seen as a ‘dynamic construct’ or a ‘continuum’, in which sharing is exhibited to varying degrees and in which physicians can make moral deliberations to arrive at the best possible decision, be it consensual or coerced [17]. A very recent publication on SDM criticises researchers' (former) focus on ‘*when* SDM is needed or applicable’ [68] and suggests focusing instead on ‘*how* best to accomplish SDM across a variety of contexts’ [69]. In the context of mental healthcare, the concept of shared responsibility and ‘*shared risk taking*’ was suggested as a constructive way to look at moral dilemmas according to risk‐taking and SDM in this context [70]. Additionally, ‘*supported decision making’*, as largely integrated into the ‘*Open Dialogue’* model [71, 72, 73], involving the therapeutic engagement of trusted supports to enhance an individual's capacity in the decision‐making process and enabling him or her to retain autonomy in life decisions, has emerged as a valuable alternative to the older ‘*substitute decision making’*. It could help health professionals to further strive for care that remains guided by a patient's will and preferences [74] and that is ‘*as shared as possible’* with the patient himself [17]. Lastly, ‘psychiatric advance directives’ may provide an opportunity for patients to outline their preferences about their mental health treatment in the event that they become unable to make such decisions for themselves in the future and may serve as a way to further protect a patient's autonomy over treatment decisions [75].

Also, there is justifiable criticism of the fact that stigma and self‐stigma still seem to remain high in mental healthcare and have a great impact on information flows, the offer of choice, power (im)balances and patients' self‐efficacy to participate. Further work seems needed to eradicate all stigma and self‐stigma in this setting and to ensure that patients, carers and clinicians are properly equipped with the knowledge and skills to provide reciprocal support, increase the flow of communication and allow patients to take greater ownership of their illness and its treatment. As Verwijmeren et al. stated earlier, *‘there is much talk of inclusion and diversity worldwide, and these themes are just as relevant for people with mental health problems*.’ [17]. The ‘*good physician‐patient relationship*’ has previously been identified as being part of the value ‘*Security*’, being particularly relevant in SDM processes with more ‘vulnerable’ patients, such as mental healthcare patients [65]. Taking into account the existing vulnerability and lack of power patients currently experience in mental healthcare [76], and the increasing data confirming patients' need for supportive healthcare providers and trusting relationships to foster their autonomy in SDM [77, 78], the systematic integration of this relational aspect in SDM, including physicians' investment in the build‐up of a trusting relationship, seems underrecognised but warranted, amongst other factors, to further support patients' participation in this setting [76]. If we can acknowledge that mental healthcare is a joint, continuous and sometimes arduous journey, we will discover the true therapeutic power and effectiveness of SDM in mental healthcare [17].

### 4.4 Strengths and Limitations

To our knowledge, this is the first review that specifically compares patient‐reported barriers and facilitators to participation in SDM in adult patients with mental illness in the setting of mental healthcare. We adopted a broad definition of ‘mental healthcare’, which resulted in a heterogeneous mix of mental illness diagnoses, illness severity (mild, moderate and severe) and healthcare settings (inpatient, outpatient and primary and secondary care) in our sample. Although we recognise that the included settings and diagnoses were characterised by important differences, we did observe a great consistency in patient experiences across the included studies as well as great similarities with findings in the most recent literature on SDM in mental healthcare [17]. This suggests that we have correctly identified the key factors influencing patients' participation within this (broad) setting and supports the generalisability of the findings across different mental healthcare settings. We are convinced that our focus on *patient*‐*reported* data is a strength. It helped us to improve our insights into the current implementation issues of SDM from the perspective of the target group, including the ‘blind spots’, factors that other stakeholders are not aware of because of lack of disease experience.

We do realise, however, that focusing on the perspective of one stakeholder group includes the risk of missing out on broader factors. The majority of barriers and facilitators patients mentioned were related to the ‘*microlevel*’, referring to the level of factors that are connected to the patient and physician, and the involvement of the patient's relatives [79]. Although this finding is in line with recent data on explanations for differences in the degree of SDM, as experienced by patients [79], it does not capture the full range of earlier‐identified barriers and facilitators to SDM, such as physician‐ and context‐related factors, as well as factors linked to the meso‐ or macrolevel [17]. Direct enquiry analyses also risk missing out on unconsciously present factors in the interrogated group, such as problems of misconceptions or other biases. The probable bias of ‘self‐stigma’, for example, was never explicitly captured by patients in the included records but seemed to be recognisable through deeper reading. Patients' reporting on SDM is subjective and coloured by prior (life or healthcare) decisional experiences, patient–physician dynamics, self‐image and cultural norms, as well as by knowledge of and familiarity with the concept of SDM, which may be lacking, incorrect or incomplete. Our sample contained an overrepresentation of papers originating from Western countries (12/13 originating from Western countries, 1 study originating from Japan). Although this distribution is in line with the global distribution pattern of research on SDM, it should kept in mind while interpreting the results, as differences in patient perceptions of SDM across other regions and cultures are present [80] and may not be represented in our results.

Lastly, we realise that the quantification of qualitative data requires contextualisation since the presence, absence or frequency of reporting does not necessarily say something about the (ir)relevance of the reported factor. We tried to offer this contextualisation as much as possible. We do believe, however, that the quantification helped to identify patterns in the gathered data.

## 5 Conclusion

We conclude that people with mental illness have, overall, a great need and motivation to be well‐informed and to be engaged in the decision‐making process but still encounter important barriers such as lack of choice, stigma, power imbalances and disease burden. Nevertheless, patients' belief in the importance of their own role and active participation seems to be greater in this setting than in patients without mental illness and could be considered protective against the identified barriers. Despite the complexities that will remain inherent to the context of mental healthcare, we should continue to emphasise the importance of individual empowerment through SDM, as this has been shown to improve patient outcomes most effectively.

We hope this study will help to contribute to the development of valuable SDM strategies in mental health care and may ultimately serve one of the main priorities in contemporary SDM research, which is the ethical imperative of greater equality and inclusion of diverse patient groups in different SDM settings.

## Author Contributions

**Lien Mertens:** conceptualisation, writing – original draft, formal analysis, methodology. **Joris Vandenberghe:** writing – review and editing. **Geertruida Bekkering:** writing – review and editing. **Karin Hannes:** writing – review and editing, supervision. **Nicolas Delvaux:** writing – review and editing. **Pieter Van Bostraeten:** writing – review and editing. **Jasmien Jaeken:** writing – review and editing. **Bert Aertgeerts:** supervision, writing – review and editing, conceptualisation. **Mieke Vermandere:** supervision, writing – review and editing.

## Conflicts of Interest

The authors declare no conflicts of interest.

## Supporting information

Supporting information.

## Data Availability

The authors have nothing to report.
