# Supplementary material for: Navigating Power Imbalances and Stigma in Mental Healthcare. Patient‐Reported Barriers and Facilitators to Participation in Shared Decision‐Making in Mental Health Care, a Qualitative Meta‐Summary
Source: Health Expect. 2025 Apr 7;28(2):e70239. doi: 10.1111/hex.70239 (PMC11974453; doi:10.1111/hex.70239)
Supplement: Supplementary file 1 — Supporting information. [file HEX-28-e70239-s001.docx]

APPENDIX

**METHODOLOGY**

1. **Search strategy - Medline**

| **Concept 1: Decision-Making** | “Decision Making”[Mesh:NoExp] OR “Decision Making"[tiab] OR "Decision Making, Shared"[Mesh] OR “SDM”[tiab] OR “sharing decision*”[tiab] OR “shared decision*”[tiab] OR “decision process*”[tiab] OR “choice making”[tiab] OR “patient choice*”[tiab] OR “patient decision*”[tiab] OR “preference sensitive”[tiab] |
| --- | --- |
| **Concept 2: Patient Participation** | "Patient Participation"[Mesh] OR "Patient Participation"[tiab] OR “Patient Engagement”[tiab] OR “Patient involvement”[tiab] OR "Patient Preference"[Mesh] OR "Patient Preference*"[tiab] OR "Physician-Patient Relations"[Mesh] OR "Physician-Patient Relation*"[tiab] OR “Patient-Physician Relation*”[tiab] OR “Doctor-patient relation*”[tiab] OR “Patient-doctor relation*”[tiab] OR “Practitioner-Patient Relation*”[tiab] OR “Patient-Practitioner Relation*”[tiab] OR "Refusal to Participate"[Mesh] OR "Refusal to Participate"[tiab] |
| **Concept 3: Patient Perceptions** | “patient report*”[tiab] OR “patient identified”[tiab] OR “patient related”[tiab] OR “patient-specific”[tiab] OR Perception*[tiab] OR Perspective*[tiab] OR View*[tiab] OR Experience*[tiab] OR “Opinion”[tiab] OR "Attitude"[Mesh:NoExp] OR “Attitude*”[tiab] OR "Attitude to Health"[Mesh] OR "Health Knowledge, Attitudes, Practice"[Mesh:NoExp] OR “Patient Satisfaction”[Mesh] OR "Patient Satisfaction"[tiab] OR belief*[tiab] OR believ*[tiab] OR "Motivation"[Mesh:NoExp] OR "Motivation*"[tiab] OR “Expectation*”[tiab] OR barrier*[tiab] OR obstacle*[tiab] OR block*[tiab] OR difficult*[tiab] OR problem*[tiab] OR hinder*[tiab] OR facilitat*[tiab] OR enabl*[tiab] OR promot*[tiab] OR help*[tiab] OR encourag*[tiab] |
| **Concept 4: Study Design** | "Surveys and Questionnaires"[Mesh] OR survey*[tiab] OR questionnaire*[tiab] OR "Interviews as Topic"[Mesh] OR interview*[tiab] OR "Focus Groups"[Mesh] OR “focus group*”[tiab] |

1. **Eligibility criteria**

***Type of participants***Families, carers, parents and advocates of patients that were chosen by patients to be involved in the SDM process, or acting as substitute decision makers, were excluded as possible participants since the role they take in the process differs from that of the patient, and so do the experienced barriers and facilitators associated to this role(1–4). Patients facing an unexpected life-threatening emergency needing immediate life-saving care were evenly excluded, following the latest consensus on the inappropriateness of SDM in these circumstances(5). Age of patients was restricted to 18 years or older so studies solely focusing on reporting by children or minor adolescents were excluded, since we know SDM in paediatric~~s~~ **patients** is characterized by its own challenges(6). Studies including patients with cognitive impairment or a (temporary) lack of mental capacity such as unconscious or comatose patients prohibiting their participation in SDM were evenly excluded. Also simulated patients or studies evaluating patients’ perspectives in hypothetical scenario settings (e.g. discrete choice experiments) were excluded.

***Types of outcomes***We wanted studies to be specifically focused on factors influencing patient’s *actual* involvement, so we excluded studies solely focusing on patient’s *preferred* involvement (syn.: participation preference, decisional role preference, decisional control preference, willingness/desire to participate, etc.) for this reason. We included studies reporting on patient characteristics (e.g. age, ethnicity, level of education…) only if these characteristics had been explicitly reported by patients as hindering or facilitating factors to their involvement, and not when these characteristics had been statistically associated to levels of involvement (see further).

***Types of studies***Data collection had to be based on patient surveys, (semi-)/(un-)structured interviews, focus groups or work groups. Interventional studies, exploring patients’ perspective on the characteristics of the intervention (feasibility, attractiveness, etc) as part of the evaluation process of a (decision support) intervention, were excluded since we wanted to focus on intervention-independent barriers and facilitators. Data originating from patient surveys were included provided that the primary focus of the survey was to identify facilitators and barriers and to quantify its relevance using Likert scales or other rating modalities. Data needed to be collected through direct enquiry with patients, so studies reporting associations between specific factors (e.g. patient characteristic) and patient involvement, brought up by the researcher based on statistical analysis, were excluded. Studies using mixed qualitative data collection tools (e.g. combining participant observations and patient interviews) were included, but only the data originating from direct enquiry of patients (e.g. patient interview data) were included for analysis. Articles not presenting results from empirical studies and grey literature were excluded. Reviews were excluded because we assumed that our search includes any relevant included study in reviews.

1. **Study identification and data extraction**

We used a ‘*mixed purposeful sampling strategy*’ to refine the selection and analysis process of the original set of eligible articles in the larger review (n=209)(Mertens et al, PEC, 2024).
For additional clarification, we refer to this paper.

We coded the eligible articles (n=209) based on two factors: 1) the ‘conceptual overlap’, the degree of overlap between the research question of the article and that of our review and 2) the information richness or ‘data density’ of the article, reflecting the quality of the article(7). All eligible studies were attributed a code ranging from 1 to 3, reflecting the degree of correspondence to the aforementioned factors and representing a ‘low’, ‘medium’ and ‘high relevance’ respectively. Articles that asked more broad questions about making treatment decisions or articles that were only loosely framed as being about SDM, were considered as being of (too) low relevance (‘code 1’) and rejected for further analysis. Articles with a medium (‘code 2’) or high relevance (‘code 3’) were withheld for further *intensity sampling*, *maximum variation sampling* and *confirming/disconfirming case sampling*(8,9).

***Data synthesis****1) Step 1: Intensity sampling*
We selected all articles identified as highly relevant in the previous phase of purposeful sampling and analysed these to define the first set of categories, which we defined inductively and independently (ML, KT), inspired by earlier data on this topic(10). From that point on, we aimed to deepen the categories further by investigating the relation between, and variation within, the different categories by means of maximum variation sampling.

***2)*** *Step 2: Maximum variation sampling*We exhaustively sought for studies that could complement the first findings by highlighting different aspects of our topic of interest, by searching for studies that *‘differed in their study designs on several dimensions*’(9). For this review, this considered studies that explored patients’ perspectives within a particular patient population group or decisional setting, and highlighted (sub)categories that had not yet been defined within the first step. This sampling and analysis were done by the main researcher (ML), within the set of articles that we had attributed a ‘code 2’. Based on the collected data from the intensity sampling and maximum variation sampling steps, a preliminary framework was constructed.

*3) Step 3: Confirming/disconfirming case sampling*Lastly, we aimed to refine and test the framework by selecting articles that ‘did not fit [the emerging patterns]’(9), representing a ‘confirming/disconfirming case sampling’ approach. More specifically, articles that brought up (sub)categories that were contradictory to the ones we defined in the earlier sampling steps, were selected and further considered for in-depth analysis.

**References:**

1. Tilse C, Willmott L, Wilson J, Feeney R WB. Operationalizing legal rights in end-of- life decision-making: A qualitative study. Palliat Med. 2021;35(10):1889–96.

2. Hutchison LA , Raffin-Bouchal DS, Syme CA, Biondo PD SJ. Readiness to participate in advance care planning: A qualitative study of renal failure patients, families and healthcare providers. Chronic Illn. 2017;13(3):171–87.

3. Laidsaar-Powell, R.; Butow, P.; Bu, S.; Charles, C.; Gafni, A.; Fisher, A.; Juraskova I. Family involvement in cancer treatment decision-making: A qualitative study of patient, family, and clinician attitudes and experiences. Patient Educ Couns. 2016;99(7):1146–55.

4. Laidsaar-Powell R, Butow P, Bu S, Fisher A JI. Attitudes and experiences of family involvement in cancer consultations: a qualitative exploration of patient and family member perspectives. Support Care Cancer. 2016;24(10):4131–40.

5. van der Horst D.E.M., Garvelink M.M., Bos W.J.W et al. For which decisions is Shared Decision Making considered appropriate? - A systematic review. Patient Educ Couns. 2023;106:3–16.

6. Boland L, Graham ID, Légaré F et al. Barriers and facilitators of pediatric shared decision-making: a systematic review. Implement Sci. 2019;14(7).

7. Carroll C, Booth A et al. Should we exclude inadequately reported studies from qualitative systematic reviews? An evaluation of sensitivity analyses in two case study reviews. Qual Heal Res. 2012;22:1425–34.

8. MQ P. Qualitative Evaluation and Research Methods (2nd Ed).

9. H S. Purposeful sampling in qualitative research synthesis. Qual Res J. 2011;11:63–75.

10. Joseph-Williams N, Elwyn G EA. Knowledge is not power for patients: A systematic review and thematic synthesis of patient-reported barriers and facilitators to shared decision making. Patient Educ Couns. 2014;94:291–309.
